# SYK Targeting Represents a Potential Therapeutic Option for Relapsed Resistant Pediatric *ETV6-RUNX1* B-Acute Lymphoblastic Leukemia Patients

**DOI:** 10.3390/ijms20246175

**Published:** 2019-12-07

**Authors:** Valentina Serafin, Elena Porcù, Giuliana Cortese, Elena Mariotto, Giulia Veltri, Silvia Bresolin, Giuseppe Basso, Benedetta Accordi

**Affiliations:** 1 Istituto di Ricerca Pediatrica Città della Speranza, 35127 Padova, Italy; silvia.bresolin@unipd.it (S.B.); giuseppe.basso@unipd.it (G.B.); benedetta.accordi@unipd.it (B.A.); 2 Laboratory of Pediatric Oncohematology, Department of Woman’s and Child’s Health, University of Padova, 35128 Padova, Italy; elena.porcu@unipd.it (E.P.); elena.mariotto@unipd.it (E.M.); giulia.veltri@phd.unipd.it (G.V.); 3 Department of Statistical Sciences, University of Padova, 35121 Padova, Italy; gcortese@stat.unipd.it

**Keywords:** leukemia, SYK, relapse, entospletinib

## Abstract

The presence of the chromosomal rearrangement t(12;21)(*ETV6-RUNX1*) in childhood B-acute lymphoblastic leukemia (B-ALL) is an independent predictor of favorable prognosis, however relapses still occur many years later after stopping therapy, and patients often display resistance to current treatments. Since spleen tyrosine kinase (SYK), a cytosolic nonreceptor tyrosine kinase interacting with immune receptors, has been previously associated with malignant transformation and cancer cell proliferation, we aimed to assess its role in *ETV6-RUNX1* cell survival and prognosis. We evaluated the effects on cell survival of three SYK inhibitors and showed that all of them, in particular entospletinib, are able to induce cell death and enhance the efficacy of conventional chemotherapeutics. By using reverse phase protein arrays we next revealed that activated SYK is upregulated at diagnosis in pediatric *ETV6-RUNX1* patients who will experience relapse, and, importantly, hyperactivation is maintained at a high level also at relapse occurrence. We thus treated primary cells from patients both at diagnosis and relapse with the combination entospletinib + chemotherapeutics and observed that SYK inhibition is able to sensitize resistant primary cells to conventional drugs. Entospletinib could thus represent a new therapeutic option supporting conventional chemotherapy for relapsed *ETV6-RUNX1* patients, and these evidences encourage further studies on SYK for treatment of other relapsed resistant acute lymphoblastic leukemia (ALL) subgroups.

## 1. Introduction

B-acute lymphoblastic leukemia (B-ALL) represents the most common malignancy in childhood. These patients are characterized by recurrent genetic alterations, including aneuploidy and structural rearrangements that commonly result in the expression of chimeric fusion genes (e.g., *ETV6-RUNX1*, *TCF3-PBX1*, *BCR-ABL1* and rearrangements of *KMT2A*) or in deregulated genes by juxtaposition to antigen receptor gene loci [1]. Although the presence of *ETV6-RUNX1* translocation is usually associated with a good prognosis, relapse still occurs in about 10% of patients many years after the end of therapy [2], and a considerable number of patients also experience a second relapse, being completely refractory to all available therapeutic agents (i.e., blinatumomab or epratuzumab) [3,4]. So far, little is known about deregulated genes or pathways that could predispose patients to relapse; however, recently Perova et al. [5] reported the relevance of spleen tyrosine kinase (SYK) activation in sustaining the growth of multiple high-risk (HR) B-ALL subtypes, showing that SYK inhibitors, such as fostamatinib, potently reduce the disease burden in mice xenotransplant studies, suggesting that SYK inhibitors may improve the outcome for HR and relapsed B-ALL patients [5]. SYK is a cytosolic nonreceptor protein tyrosine kinase that contains a C-terminal kinase domain and tandem N-terminal SH2 domains that bind the phosphorylated immunoreceptor tyrosine-based activation motifs (ITAMs) of immune receptors such as the B-cell receptor. In normal B-lymphocytes the general activation of the SYK pathway is mostly initiated by phosphorylation by SRC family kinases of ITAMs tyrosine residues that triggers the activation of SYK and its direct binding to other proteins such as phospholipase Cγ (PLCγ) families, the p85α subunit of phosphoinositide 3-kinase (PI3K), as well as leukocyte protein 76 (SLP76) and SLP65 [6]. These direct binding partners activate downstream signaling components, which trigger various cellular processes, including maturation of pro-B into pre-B cells, migration, adhesion, innate immune recognition and autoimmunity. Moreover, SYK has been described as having a role in malignant transformation of mature B cells, leading to various forms of B-cell lymphoma and B-cell chronic lymphocytic leukemia [7]. All these evidences prompted us to investigate the role of SYK in *ETV6-RUNX1* cell survival and prognosis, trying to elucidate molecular mechanisms responsible for drug resistance and relapse occurrence in this otherwise good-prognosis B-ALL subtype.

## 2. Results

**Figure 1:**
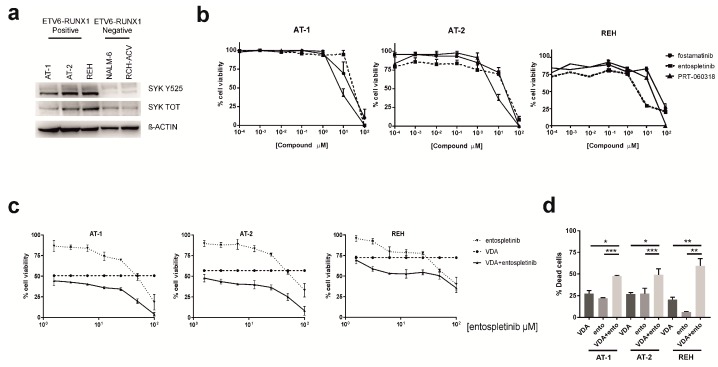
SYK inhibition in *ETV6-RUNX1* cell lines enhances the efficacy of conventional chemotherapeutics. (**a**) Western blot analysis for SYK Y525 and its total form in *ETV6-RUNX1*-positive (AT-1, AT-2 and REH) and negative (*NALM-6* and *RCH-ACV*) cell lines. (**b**) Cell viability measured by MTT test of *ETV6-RUNX1* cell lines treated for 72 h with SYK inhibitors. All experiments were performed at least three times, and data are represented as mean ± SEM. (**c**) Reduction of cell viability, determined by MTT test, in AT-1, AT-2 and REH cells after 48 h of treatment with entospletinib and 1 unit of VDA (corresponding to a cocktail of 1 nM VCR, dex and AraC respectively) alone or in combination (*n* = at least three for all experiments). Results are presented as means ± SEM. (**d**) Increased cell death determined by Annexin V/PI staining after 48 h of treatment. AT-1, AT-2 and REH cells were treated with 1 unit of VDA and 50 μM of entospletinib (ento) alone or in combination. The percentage of dead cells, defined as the total of Annexin V^+^/PI^−^, Annexin V^+^/PI^+^ and Annexin V^−^/PI^+^, was established after normalizing on DMSO-treated cells. Paired *t* test; * *p* ≤ 0.05, ** *p* ≤ 0.01; *** *p* ≤ 0.001; *n* = 3 for all experiments. Results are presented as means ± SEM. VDA and ento concentrations used in these experiments were selected on the basis of MTT test results, by choosing the ones most able to reduce cell viability in combination.

In order to assess SYK expression and activity in *ETV6-RUNX1* leukemias, as a first step we evaluated three *ETV6-RUNX1* cell lines (AT-1, AT-2 and REH) and two non-*ETV6-RUNX1* ones (NALM-6 and RCH-ACV) (Figure 1a). Interestingly, the active form of SYK Y525 was detectable in all the three *ETV6-RUNX1* cell lines. Thus, to understand the role of activated SYK in sustaining cell survival, we evaluated the effects of SYK inhibition in the three *ETV6-RUNX1* cell lines. After 72 h of treatment with the SYK inhibitors entospletinib, fostamatinib and PRT-060318, cell viability was efficiently decreased (Figure 1b). We verified by phosphoflow that, after 1 h of treatment, all the three selected SYK inhibitors were able to significantly decrease SYK activation (Figure S1). We next treated the three *ETV6-RUNX1* cell lines with the conventional ALL chemotherapeutics dexamethasone (dex), cytarabine (AraC), vincristine (VCR), daunorubicine (dauno), and L-asparaginase (L-Asp) (Sigma-Aldrich) for 48 h. We considered as resistant cell lines with a GI_50_ value ≥ 1 μM and/or cells not displaying a complete reduction of viability at the higher drug concentrations, thus all the three cell lines turned out to be resistant to dex and AraC, and only AT-1 and AT-2 to VCR (Figure S2). We thus evaluated the potential of SYK inhibition to overcome drug resistance by combining each one of the three SYK inhibitors with dex, AraC or VCR alone. The best synergistic effect, proved by the lowest values of combination index (CI), was obtained with entospletinib (Table S1), thus we decided to support entospletinib efficacy with further experiments. To best mimic the therapeutic protocol, we set up a VCR-dex-AraC cocktail (VDA) and treated AT-1, AT-2, and REH for 48 h with VDA alone or in combination with entospletinib. As expected, we observed a marked decrease in cell viability with the combination VDA + entospletinib compared to VDA or entospletinib alone (Figure 1c), with a synergistic effect confirmed by CI values reported in Table S2. Annexin V/PI staining of treated *ETV6-RUNX1* cell lines also demonstrated the ability of VDA to significantly induce more cell death when combined with SYK inhibition (Figure 1d). Moreover, we observed that in AT-1 cells, the inhibition of SYK by entospletinib generally downregulates the mTOR pathway (Figure S3), as already described in follicular lymphoma [8] and B-ALL [9] cells.

**Figure 2:**
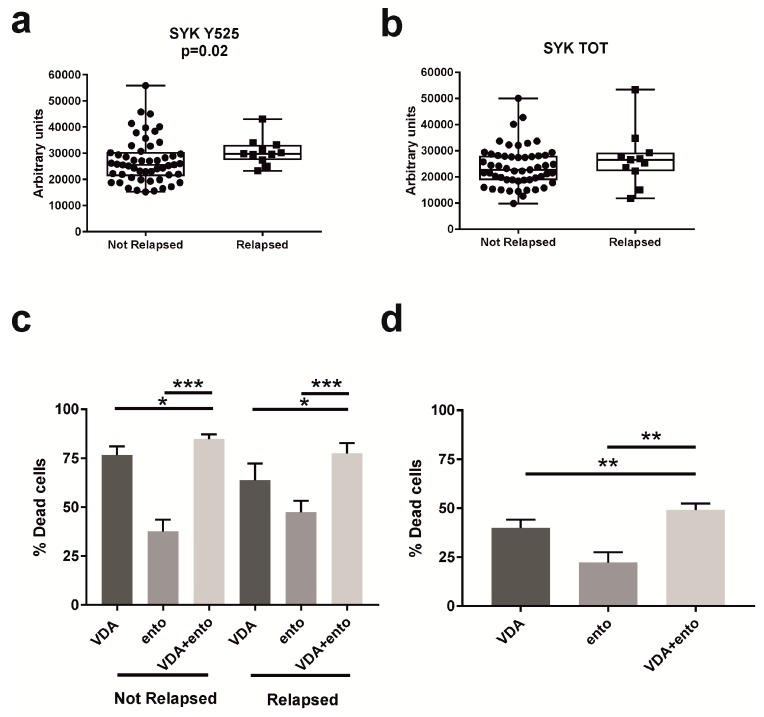
SYK inhibition sensitizes resistant primary cells to conventional drugs. (**a**) SYK Y525, measured by RPPA analysis, is upregulated in patients who relapsed (Relapsed, *n* = 11, median 29,756.5 AU) compared to patients who did not relapse (Not Relapsed, *n* = 53, median 25,578.5 AU) (unpaired *t* test with Welch’s correction, *p* = 0.02). (**b**) The total form of SYK is not differentially expressed between the two groups of patients (Not Relapsed *n* = 53, median 22,702 AU; Relapsed *n* = 11, median 26,511 AU). Results are presented as median ± max to min value. (**c**) Entospletinib decreases the cell viability of *ETV6-RUNX1* primary samples at diagnosis. Annexin V/PI staining of primary cells from *ETV6-RUNX1* patients at diagnosis (Not Relapsed *n* = 12, Relapsed *n* = 5), treated with VDA (1000 units, corresponding to a cocktail of 1 μM VCR, dex and AraC, respectively) and entospletinib (ento, 100 μM) alone or in combination for 48 h. The percentage of dead cells, defined as the total of Annexin V^+^/PI^−^, Annexin V^+^/PI^+^ and Annexin V^−^/PI^+^, was established after normalizing cells on DMSO-treated cells. Paired *t* test; * *p* ≤ 0.05, ** *p* ≤ 0.01; *** *p* ≤ 0.001. Results are presented as means ± SEM. (**d**) Entospletinib decreases the cell viability of *ETV6-RUNX1* primary samples at relapse. Annexin V/PI staining of primary cells from *ETV6-RUNX1* patients at relapse (*n* = 5), treated with VDA (1000 units) and entospletinib (ento, 100 μM) alone or in combination for 48 h. The percentage of dead cells, defined as the total of Annexin V^+^/PI^−^, Annexin V^+^/PI^+^ and Annexin V^−^/PI^+^, was established after normalizing cells on DMSO-treated cells. Paired *t* test; * *p* ≤ 0.05, ** *p* ≤ 0.01. Results are presented as means ± SEM.

These results prompted us to evaluate the levels of expression and phosphorylation of SYK in a cohort of 64 pediatric *ETV6-RUNX1* patients at diagnosis. By using reverse-phase protein arrays (RPPA) [10], we compared SYK expression and activation between relapsed (*n* = 11) and nonrelapsed (*n* = 53) patients through an unpaired *t* test with Welch’s correction. From this analysis we identified the hyperactivation of SYK, phosphorylated in Y525, at diagnosis in patients who will experience relapse (*p* = 0.02) (Figure 2a). Total SYK (Figure 2b) and *SYK* mRNA (Figure S4) were not differentially expressed between the two subgroups of patients.

In order to confirm that in patient samples, SYK inhibition is able to reverse chemoresistance, DMSO-frozen primary cells at diagnosis from 17 patients (patients who did not relapse, *n* = 12; relapsed patients, *n* = 5), were thawed, seeded and treated for 48 h with VDA and entospletinib alone or in combination. Both patients that relapsed and did not relapse responded to entospletinib, although the response to SYK inhibition tends to be higher in relapsed patients compared to nonrelapsed patients (Figure 2C). More interestingly, in patients that relapsed, the addition of entospletinib to VDA significantly increased the percentage of dead cells compared to VDA (*p* = 0.05) or to entospletinib (*p* = 0.0003) alone, highlighting the ability of SYK inhibition to sensitize leukemia cells to conventional drugs. Notably, also in patients that did not relapse, we observed an increase of cell death when primary leukemia cells are treated with entospletinib + VDA compared to VDA (*p* = 0.03) or to entospletinib (*p* < 0.0001) alone (Figure 2c). Thus, patients that are more prone to relapse will benefit from entospletinib treatment that is able to turn them sensitive to conventional chemotherapeutic drugs as much as patients that will not relapse. Finally, in order to evaluate whether SYK could be a potential therapeutic target at relapse, by phosphoflow analysis we evaluated SYK Y525/526 in 17 *ETV6-RUNX1* primary samples: eight diagnosed patients that relapsed and nine unmatched relapses; and we observed that SYK Y525/526 phosphorylation is maintained at a high level at the moment of relapse (Figures S5 and S6). Thus, to understand if SYK inhibition could be able to decrease the viability of relapsed cells, we treated five *ETV6-RUNX1* relapsed samples with VDA and entospletinib alone or in combination. Again, the combination of entospletinib + VDA significantly increased the percentage of dead cells compared to VDA (*p* = 0.0015) or to entospletinib (*p* = 0.0085) alone (Figure 2d).

## 3. Discussion

Our results reveal that tyrosine kinase SYK activation plays a pivotal role in sustaining proliferation and survival of aggressive pediatric *ETV6-RUNX1* B-ALL cells, and it can be targeted in order to reverse drug resistance. Notably, SYK inhibition resulted in the ability to synergize with conventional ALL drugs to induce leukemia cells’ deaths at relapse. This is completely in agreement with previously reported evidences on the role of SYK in B-cell malignancies [5,11,12], but our results point to the potential promising impact of inhibiting SYK to treat relapsed resistant pediatric patients. Notably, the evidence that also patients with lower active SYK respond to entospletinib has already been observed [13] and could be explained by the ability of SYK inhibitors to target also other kinases such as SRC, c-KYT and FLT3. However, this result is not affecting the therapeutic potential of adding entospletinib to conventional chemotherapy, since patients that will relapse will benefit from this novel approach to turn them more sensitive to conventional chemotherapeutic drugs and to eventually prevent relapse.

In this paper we evaluated the efficacy of SYK inhibition only in *ETV6-RUNX1* ALL since this subgroup’s results are particularly interesting due to its usually good prognosis, but with patients experiencing relapse many years later after stopping therapy, however, this does not exclude that other pediatric-relapsed ALL subtypes could benefit from the use of SYK inhibitors. This paper thus encourages further studies on the promises of SYK inhibition in association with conventional chemotherapy for treatment of relapsed pediatric ALL patients not responding to current available strategies.

## 4. Materials and Methods

### 4.1. Cell Lines and Primary Samples

Human leukemia cell line REH was purchased from DSMZ and authenticated by short-tandem repeat profiling (Biogem, Ariano Irpino (AV), Italy). Notably, the REH cell line is resistant to dex due to a lack of functional glucocorticoid receptor [14]. AT-1 and AT-2 cells were a kind gift of the group of Prof. Pieter Van Vlierberghe (Ghent University, Ghent, Belgium). Cells were cultured in RPMI 1640 (GIBCO, Thermo Fisher Scientific, Waltham, MA, USA) with 10% (REH) or 20% (AT-1 and AT-2) fetal bovine serum (FBS; GIBCO), glutamine (2 mM/L; GIBCO), penicillin (100 U/mL; GIBCO) and streptomycin (100 mg/mL) (GIBCO), and maintained at 37 °C in a humidified atmosphere with 5% CO_2_. All cell lines were periodically tested for mycoplasma infection.

B-ALL patients’ samples were obtained after informed consent, following the tenets of the Declaration of Helsinki. This study was approved by the local ethical committee “Comitato Etico per la Sperimentazione, Azienda Ospedaliera di Padova”, according to institutional guidelines and Declaration of Helsinki principles (date of approval 13/10/2000 protocol number 257 bisP). All samples were obtained at the time of diagnosis before treatment, or at relapse, after Ficoll–Hypaque (Pharmacia Ltd., Uppsala, Sweden) separation of mononuclear cells. Mononuclear cells were frozen as viable cells in FBS and 10% DMSO and stored in liquid nitrogen. Once thawed, cells were cultured in AIM-V tissue culture medium (GIBCO) with 10% FCS (GIBCO), glutamine (2 mM/L; GIBCO), penicillin (100 U/mL; GIBCO), streptomycin (100 mg/mL; GIBCO).

### 4.2. SYK Inhibitors

Entospletinib is actually evaluated in several clinical trials (i.e., phase 1b/2 for adults with relapsed or refractory ALL in association with vincristine and dexamethasone #NCT02404220, phase 2 for adults with relapsed or refractory hematologic malignancies #NCT01799889, phase 1b/2 for adults with relapsed or refractory B-cell non-Hodgkin lymphoma #NCT02568683), and fostamatinib has been just approved by the FDA for the treatment of adult patients with chronic immune thrombocytopenia [15], whereas PRT-060318 is a novel and highly specific SYK inhibitor not yet in clinical trial [16]. All SYK inhibitors were purchased from Selleckchem, Houston, TX, USA.

### 4.3. MTT Assay

Cell viability of cell lines treated with entospletinib, fostamatinib and PRT-060318 alone or in combination with chemotherapeutic drugs was assessed by MTT ((3-(4,5- dimethylthiazol-2-yl)-2,5-diphenyl tetrazolium bromide) assay as previously described [10].

### 4.4. Annexin V-PI Assay

Cell death of cell lines and primary samples treated with entospletinib alone or in combination with VDA was assessed after 48 h of treatment by the AnnexinV–FLUOS staining kit (Roche, Basel, Switzerland), following the manufacturer’s instructions. Samples were analyzed by flow cytometric analysis (Cytomics FC500, Beckman Coulter, Brea, CA, USA).

### 4.5. Western Blot

SDS–polyacrylamide gel electrophoresis was performed using NuPAGE 4 to 12% bis-tris gels (Invitrogen, Thermo Fisher Scientific), and proteins were transferred to PVDF membranes by semidry transfer using NuPAGE transfer buffer (Invitrogen). Membranes were blocked in I-block 2% (Invitrogen), incubated overnight at 4 °C with primary antibodies and 1 h with the HRP-conjugated secondary antibody (PerkinElmer, Waltham, MA, USA). Bands were detected using the Alliance (Uvitec Cambridge, Cambridge, UK). The following antibodies were used: SYK Y525 (1:100 Abcam, Cambridge, UK), SYK TOT (M01A, 1:100) (Abnova, Taipei, Taiwan), mTOR S2448 (1:100, Novus Biologicals, Centennial, CO, USA), PRAS40 T246 (1:100, Thermo Fisher Scientific), S6 Ribosomal Protein S235/236 (1:100, Cell Signaling Technology, Danvers, MA, USA), eIF4EBP1 S65 (174A9, 1:100) (Cell Signaling Technology,), and β-actin (1:10,000 Sigma-Aldrich, Saint Louis, MO, USA).

### 4.6. Phospho-Flow Cytometry

AT-1, AT-2 and REH cell lines and primary B-ALL cells were harvested, fixed in 1.5% paraformaldehyde and permeabilized with 100% methanol. Samples were stored in 100% methanol at −20 °C. After recovering, they were stained by SYK Y525/526 (C87C1) primary antibody (Cell Signaling Technology) (1 µL/0.25 × 10^6^ cells), and then incubated with anti-rabbit secondary antibody conjugated with Alexafluor 488 dye (1:500, Molecular Probes, Thermo Fisher Scientific). Patients’ samples were analyzed on a Cytoflex flow cytometer (Beckman Coulter). Cell lines were analyzed on Cytomic FC500 flow cytometer (Beckman Coulter). For patients, data are presented as Median Fluorescence Intensity (MFI) of positive cells in the live-gated CD45^dim^/CD19^+^cell subpopulation. Overlay histograms were set up with FlowJo software (Tree Star Inc., Ashland, OR, USA), and heatmaps were set up with the Morpheus platform (https://software.broadinstitute.org/morpheus/).

### 4.7. RPPA

We analyzed 64 pediatric *ETV6-RUNX1* B-ALL patients at diagnosis with Reverse Phase Protein Arrays [10] for the expression of SYK Y525 (Abcam) and SYK (M01A) (Abnova) validated primary antibodies. Of this cohort, 11 patients relapsed. Samples were collected at the Pediatric Oncohematology Laboratory (University of Padova, Padova, Italy) between 2000 and 2009 and were enrolled in the AIEOP-BFM ALL2000/R2006 therapy protocol [17]. Patients stratified in the high-risk (HR) arm were excluded to avoid misleading results due to different treatment. The whole blood blast percentage for all samples was between 72% and 96%.

### 4.8. Statistical Analysis

Assessment of differentially activated or expressed SYK between relapsed and nonrelapsed patients from RPPA experiments was obtained through an unpaired *t* test with Welch’s correction.

MTT and Annexin V/PI experiments were performed at least 3 times, and data were represented as mean ± SEM. The difference between untreated and treated cells was evaluated using a paired *t* test, performed by using Prism 7 (GraphPad Software, Inc., San Diego, CA, USA). To determine the synergistic, additive, or antagonistic effects of the drug combinations from the MTT experiments, we used CalcuSyn software, which is based on the method of the combination index (CI) of Chou and Talalay [18].

## Supplementary Materials

The following are available online at https://www.mdpi.com/1422-0067/20/24/6175/s1, Table S1: Combination Index (CI) values in AT-1, AT-2 and REH cell lines. Table S2: CI values in AT-1, AT-2 and REH cell lines. Figure S1: SYK Y525/Y526 phosphorylation measured by phosphoflow. Figure S2: Sensitivity of AT-1, AT-2 and REH cells to the conventional chemotherapeutic compounds. Figure S3: Western blot for mTOR pathway in AT-1 cell line treated with entospletinib. Figure S4: *SYK* mRNA expression in *ETV6-RUNX1* patients. Figure S5: Phosphorylation of SYK Y525/526 in primary cells. Figure S6: Gating strategy used in phospho-flow cytometry analysis.
