# Supplementary material for: SYK Targeting Represents a Potential Therapeutic Option for Relapsed Resistant Pediatric ETV6-RUNX1 B-Acute Lymphoblastic Leukemia Patients
Source: Int J Mol Sci. 2019 Dec 7;20(24):6175. doi: 10.3390/ijms20246175 (PMC6940898; doi:10.3390/ijms20246175)
Supplement: Supplementary file 1 [file ijms-20-06175-s001.pdf]

Supplementary Materials

Supplementary Table S1

Combination Index (CI) values in AT-1, AT-2 and REH cell lines. CIs were calculated by Calcusyn software at the effective dose necessary to reduce the cell viability of the 50% (GI_{50}) in AT-1, AT-2 and REH cell lines at 48 hours, measured by MTT test. Synergy, additivity and antagonism are defined by a CI<1, CI=1 or CI>1, respectively. Of note, experiments with dexamethasone (dex) in REH cells were not performed since these cells are resistant due to a lack of functional Glucocorticoid Receptor[14] and with vincristine (VCR) since they showed to be highly sensitive. Cells were treated at the molar ratio (Chemio: SYK inhibitor) of 1:100 for cytarabine (AraC) or 1:10 for Dex and VCR.

**Combination Index (CI) GI_{50}**	**AT-1**	**AT-2**	**REH**
**AraC+ entospletinib**	**0.162**	**0.320**	**0.956**
**AraC+ fostamatinib**	0.329	2.255	3.077
**AraC+ PRT-060318**	0.630	0.999	0.868
**Dex+ entospletinib**	**0.082**	**0.165**	ND
**Dex+ fostamatinib**	0.290	0.006	ND
**Dex+ PRT-060318**	0.639	0.817	ND
**VCR+ entospletinib**	**0.073**	**0.321**	ND
**VCR+ fostamatinib**	0.097	0.338	ND
**VCR+ PRT-060318**	0.755	1.089	ND

Supplementary Table 2

CI values in AT-1, AT-2 and REH cell lines. CI values at the indicated concentrations of entospletinib with fixed VDA at 1 unit. Ento: entospletinib, VDA (VCR+Dex+AraC).

	**Combination Index (CI)**		
	**VDA + ento**	**VDA + ento**	**VDA + ento**
	**AT-1**	**AT-2**	**REH**
**entospletinib**			
**100 µM**	0.188	0.220	0.643
**50 µM**	0.413	0.375	0.718
**25 µM**	0.385	0.357	0.403
**12,5 µM**	0.204	0.262	0.224

Supplementary figures:

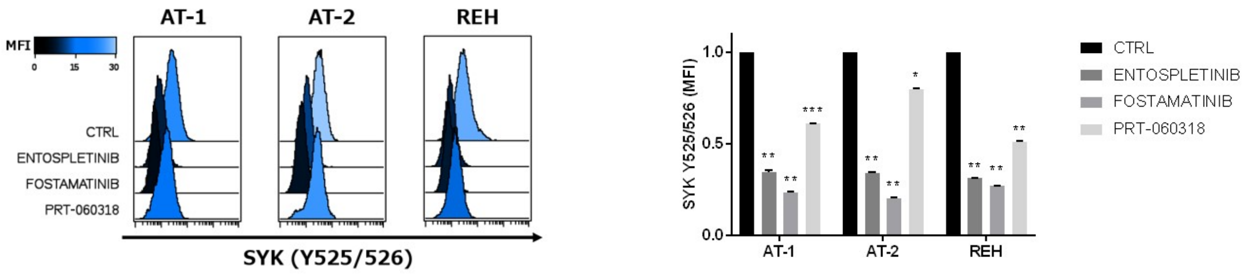

Figure S1: SYK Y525/Y526 phosphorylation measured by phosphoflow. Right panel. Reduction of SYK Y525/Y526 phosphorylation measured by phosphoflow in AT-1, AT-2 and REH cell lines after 1 hour of treatment with DMSO (CTRL) or with 5 µM SYK inhibitors. MFI= Median Fluorescence Intensity. Left panel. Fold change reduction of SYK Y525/Y526 phosphorylation measured by phosphoflow in AT-1, AT-2 and REH cell lines after 1 hour of treatment with DMSO (CTRL) or with 5 µM SYK inhibitors. Paired t test; * $p \leq 0.05$, ** $p \leq 0.01$; *** $p \leq 0.001$; n=3 for all experiments. Results are presented as means ± SEM. CTRL was set to 1.0.

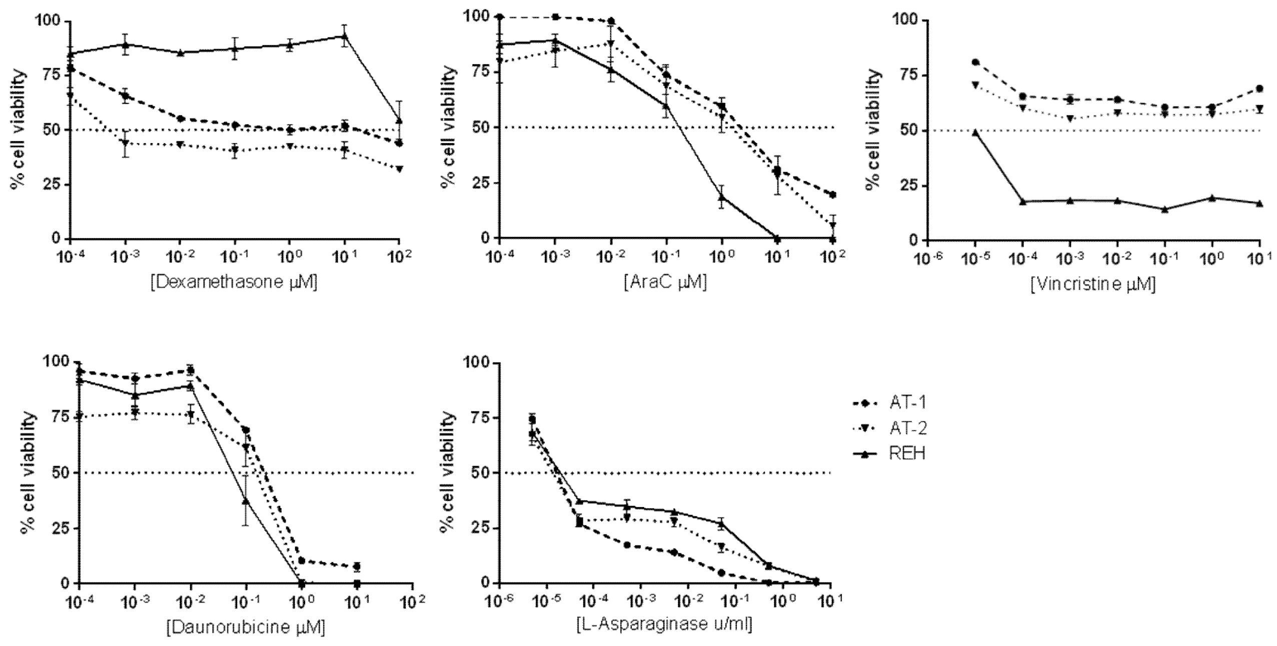

Figure S2: Sensitivity of AT-1, AT-2 and REH cells to the conventional chemotherapeutic compounds. Cell viability of AT-1, AT-2 and REH cells after treatment for 48 hours with dexamethasone, AraC, vincristine, daunorubicine and L-Asparaginase. All experiments were performed at least 3 times, and data are represented as mean ± SEM.

a

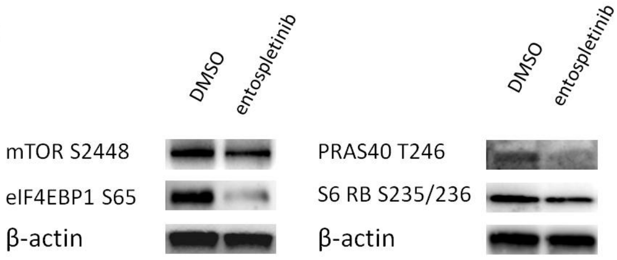

b

Antibody	DMSO	entospletinib
mTOR S2448	1,294377	0,910453
eiF4EBP1 S65	0,861115	0,276838
PRAS40 T246	0,682033	0,286682
S6 RB S235/236	0,850192	0,459063

Figure S3: Western blot for mTOR pathway in AT-1 cell line treated with entospletinib. a) Western Blot analysis for mTOR S2448, eIF4EBP1 S65, PRAS40 T246, S6 Ribosomal Protein S235/236 and β-actin in AT-1 cell line after 1 hour treatment with DMSO or with 10 μM of entospletinib. **b)** Densitometric values after normalization of antibodies signal intensity to β-actin with ImageJ software.

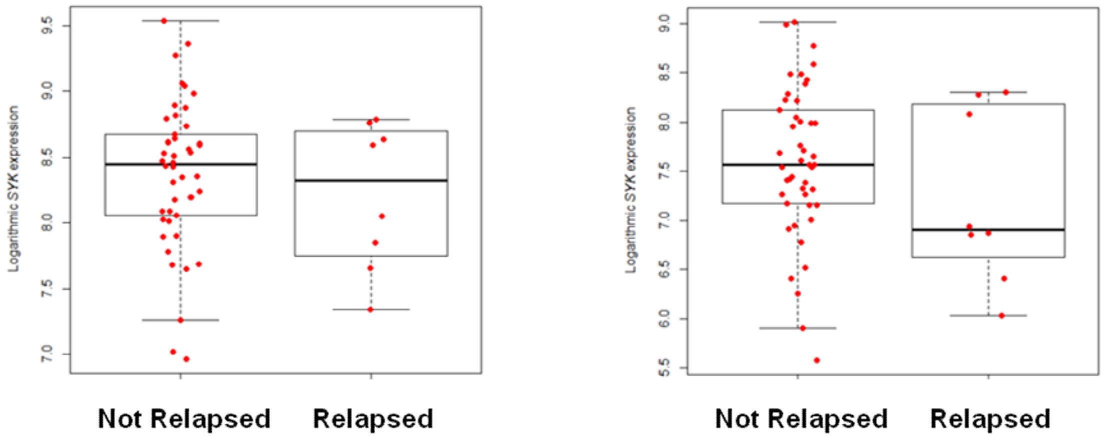

Figure S4: *SYK* mRNA expression in ETV6-RUNX1 patients. *SYK* mRNA in log2 expression measured by gene expression analysis in an independent cohort of 54 ETV6-RUNX1 B-ALL patients (n= 46 not relapsed, n= 8 relapsed) by Affymetrix HG-U133Plus 2.0 arrays. The two panels show the two different probe sets (226068_s_at and 207540_s_at) for *SYK*. The average expression of *SYK* between ETV6-RUNX1 patients who did not relapse and the ones who relapsed is not significantly different. Results are presented as mean ± SD.

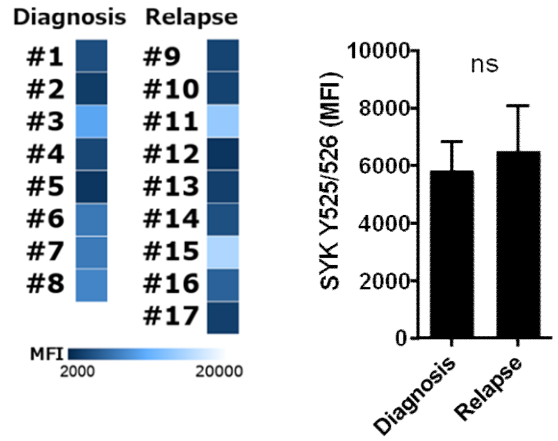

Figure S5: Phosphorylation of SYK Y525/526 in primary cells. Phosphorylation was measured by phosphoflow in primary cells from 8 diagnosis and 9 unmatched relapses of ETV6-RUNX1 patients. Data are presented as a heatmap of Median Fluorescence Intensity (MFI) of positive-cells in the live-gated cell subpopulation (left panel) or as MFI mean ± DS (right panel).

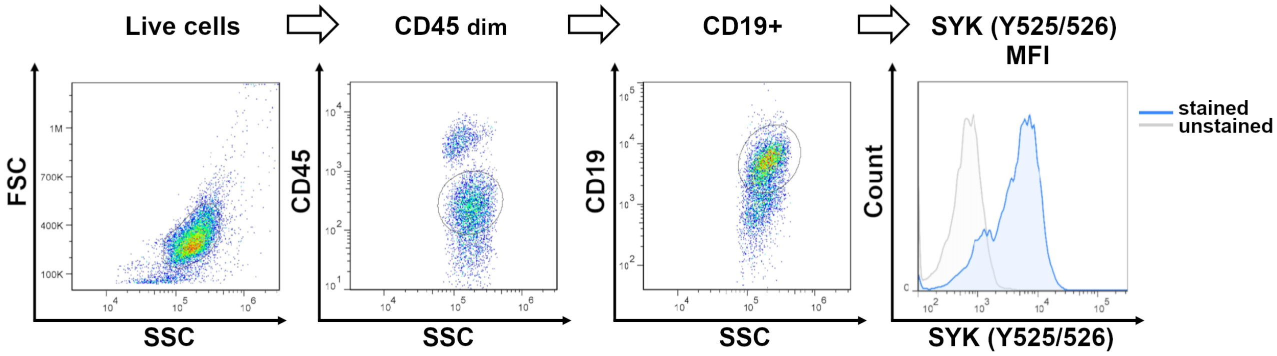

Figure S6: Gating strategy used in phospho-flow cytometry analysis. Representative image on patient #6, showing the gating strategy used for SYK (Y525/526) Median Fluorescent Intensity (MFI) detection, evaluated on live-gated $CD45^{dim}/CD19^{+}$ cell population.
